# A constraint-based framework for exploring the impact of multireaction dependencies on metabolic functions

**DOI:** 10.1038/s41540-025-00608-9

**Published:** 2025-10-23

**Authors:** Anika Küken, Damoun Langary, Angela Angeleska, Zoran Nikoloski

**Affiliations:** 1 Bioinformatics, Institute of Biochemistry and Biology, University of Potsdam, Potsdam, Germany; 2 Systems Biology and Mathematical Modeling, Max Planck Institute of Molecular Plant Physiology, Potsdam, Germany; 3 Mathematics Department, University of Tampa, Tampa, FL USA

**Keywords:** Biochemical networks, Computer modelling, Metabolic engineering

## Abstract

Metabolism operates under physico-chemical constraints that result in multireaction dependencies. Understanding how multireaction dependencies affect metabolic phenotypes remains challenging, hindering their biotechnological applications. Here, we propose the concept of a forcedly balanced complex that allows to efficiently determine the effects of specific multireaction dependencies on metabolic network functions in constrained-based models. Using this concept, we found that the fraction of multireaction dependencies induced by forcedly balanced complexes in genome-scale metabolic networks followed power law with exponential cut-off. We identified forcedly balanced complexes that are lethal in cancer but have little effect on growth in healthy tissue models. In addition, these forcedly balanced complexes are largely specific to models of particular cancer types. Therefore, multireaction dependencies resulting from forced balancing of complexes represent an innovative means to control cancers that, we argue, can be implemented via transporter engineering. The presented constraint-based approaches pave the way for using multireaction dependencies in metabolic engineering for diverse biotechnological applications.

## Introduction

Metabolism underpins all life on earth and comprises interdependent pathways that synthesize and degrade complex macromolecules, such as nucleic acids, proteins, polysaccharides, and lipids^1^. As a result, metabolism is key to conversion of nutrients into energy that fuel all biochemical reactions in living cells. These metabolic conversions take place under physico-chemical constraints, such as steady-state operation^2^ and thermodynamic feasibility^3^, which lead to pairwise dependence between reaction fluxes. The dependence between reaction fluxes arises due to the participation of metabolites in multiple reactions, and, thus, reflects the structure of the underlying metabolic networks. The availability of large-scale metabolic networks, gathering all biochemical reactions involved in metabolic conversions in individual cell types, tissues, organs, entire organisms along with their interactions in ecological communities^4–9^, has facilitated the study of the relationships between reaction fluxes at steady state^10^ and their implications for the modularity^11,12^ and control^13^ of metabolic networks.

However, recent results have shown that metabolic networks harbor functional relationships that include more than only pairs of reactions^14,15^. For instance, it has been shown that multireaction dependencies can be: (i) efficiently identified by relying on the structure of metabolic networks^14^, (ii) employed to identify modules in large-scale metabolic networks^14^, (iii) used for accurate estimation of reaction fluxes, in line with estimates resulting from labeling experiments^15^. While these findings suggest that regulation of metabolism is related to maintaining relationship between steady-state fluxes of certain reactions, they do not point at implications of imposing multireaction dependencies on the manipulation of metabolic network functions.

Building on the intricate relationship between metabolic network structure and function at steady state^14,16–18^, here we first define the concept of a balancing potential obtained by imposing balancing in particular points in the network via so-called forcedly balanced complexes. We then (i) devise an efficient procedure to determine the distribution of balancing potentials in large-scale metabolic networks, (ii) show how the balancing potential relates to concordant modules in metabolic networks, and (iii) demonstrate that it is a universal property for metabolic networks across species of all kingdoms of life. To study the implication of forced balancing to the functionality of metabolic networks, we determine points in metabolic networks of cancers and healthy tissues with differential effect of forced balancing on growth in the respective tissues. As a result, we pinpoint a means to reduce cancer growth going beyond standard manipulations of reaction fluxes and respective gene expression, such as overexpression, down-regulation, and knock-outs^19–21^. Lastly, we discuss the ways by which balancing can be imposed in metabolic networks, providing potential strategies to experimentally verify the manipulation strategies resulting from the forced balancing of specific points in metabolic networks. Therefore, our work provides a new approach for manipulation of metabolic network function, that goes beyond the well-established techniques from constraint-based modeling^22,23^, toward achieving biotechnological goals.

## Results

### Definition of forcedly balanced complexes and classification of implied balanced complexes

**Fig. 1:**
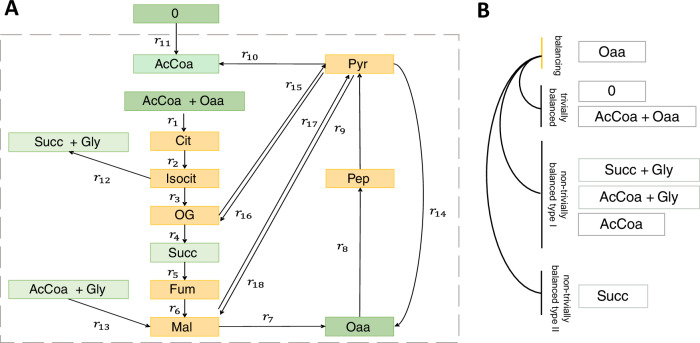
Illustration of balanced and concordant complexes along with the implications of balancing complexes. **A** Network including 11 species (AcCoa, - Acetyl-CoA, Cit – Citrate, Fum – Fumarate, Gly - Glyocalate, Isocit – Isocitrate, Mal – Malate, Oaa – Oxaloacetate, OG – Oxoglutarate, Pep – Phosphoenolpyruvate, Pyr – Pyruvate, Succ – Succinate, 0 – zero-complex), 14 complexes depicted as rectangles, and 18 irreversible reactions, ${r}_{1}-{r}_{18}$, each connecting two complexes. The network includes the zero complex that models the interaction with the environment. The dashed line shows the network boundary. Balanced complexes are shown in yellow. The three concordance modules composed of non-balanced complexes are depicted in different shades of green. **B** Implication from balancing the non-balanced complex $1\cdot {\rm{Oaa}}$.

To define the concept of a *forcedly balanced complex*, we first introduce some key concepts from stoichiometric modeling of biochemical networks. A biochemical network is composed of reactions through which biochemical species acting as substrates are transformed into products. Here each reversible reaction is split into two irreversible reactions, denoting the forward and backward direction of the corresponding reversible reaction. The biochemical network in Fig. 1A is composed of 18 irreversible reactions that transform 11 species. The set of species jointly consumed (or produced) by a reaction corresponds to a complex^24^. In other words, each complex in a biochemical network is given by a non-negative linear combinations (specified by the stoichiometry) of species in the network^24^, corresponding to the left- and right-hand side of a reaction. For instance, reaction $\rm{r}_{1}:{\rm {AcCoa}}+{\rm {Oaa}}\to {\rm{Cit}}$ leads to two complexes, ${\rm {AcCoa}}+{\rm {Oaa}}$ and ${\rm {Cit}}$. We note that the complexes are mathematical constructs derived from the left- and right-hand sides of reactions and thereby result from the biochemical structure of the metabolic networks. Also note that, complexes are sensitive to stoichiometric scaling of individual reactions. Hence, we assume a consistent use of conventional stoichiometric scaling (e.g., scaling to smallest integer values) when comparing results across models. However, complexes as defined here, are not to be confused with enzyme-metabolite or protein-protein complexes studied in biochemistry. The network can then be represented as a directed graph composed of nodes, denoting complexes, and edges, representing reactions. For example, the network of 18 reactions in Fig. 1A contains 14 complexes. The stoichiometric matrix of the network, ${\bf{N}}$, is then given by the product of the matrix, ${\bf{Y}}$, describing the species composition of complexes and the incidence matrix, ${\bf{A}}$, of the corresponding directed graph (Supplementary Fig. S1)^25,26^. In addition, the reactions are weighted by non-negative numbers which correspond to fluxes of a steady-state flux distribution, ${\bf{v}}$, which satisfies ${\bf{Nv}}={\mathbf{0}}$.

The representation of a biochemical network in terms of complexes and reactions allows a systematic evaluation of reaction flux dependencies around complexes. This representation allows us to narrow down the set of possible multi-reaction dependencies to a size that is amenable to computations^14^ and from which a rich collection of results about network dynamics and steady states have already been derived^24,27–29^. We refer to a complex $${{\rm{C}}}_{i}$$ as balanced if the sum of fluxes of its incoming reactions equals the sum of fluxes of its outgoing reactions in every steady-state flux distributions in $$ S$$. The set of reactions around complex $${{\rm{C}}}_{i}$$ is given by the indices of the non-zero entries in the $$i$$-th row, $${{\bf{A}}}^{i:}$$, of the incidence matrix $${\bf{A}}$$, and corresponds to the support of $${{\bf{A}}}^{i:}$$. Hence, the complex $${{\rm{C}}}_{i}$$ is balanced if its *activity*, given by $${{\bf{A}}}^{i:}{\bf{v}}$$, is zero for any steady-state flux distribution $${\bf{v}}$$ that the network obtains (i.e., any $${\bf{v}}$$ from $$S$$ that satisfies $${\bf{YAv}}={\bf{Nv}}={\bf{0}}$$)^17,18^. Clearly, complexes that act as sinks or sources, that only have incoming or outgoing reactions, respectively, cannot be balanced in a network without blocked reactions (i.e., reactions that carry no flux in every steady state the network supports).

Further, a complex is considered *trivially balanced* if it includes a species that appears in no other complex in the network. This result is a consequence of the balancing of species due to the steady state assumption. A balanced complex that includes species all of which appear in other complexes is termed *non-trivially balanced*. For instance, for the network in Fig. 1A, complex 1∙${\rm{AcCoa}}$ cannot be balanced as it is a sink; complexes $1\cdot {\rm{Mal}}$, $1\cdot {\rm{Pyr}}$, $1\cdot {\rm{Cit}}$, $1\cdot {\rm{Isocit}}$, $1\cdot {\rm{OG}}$, $1$∙${\rm{Fum}}$, and $1$∙${\rm{Pep}}$, which have both incoming and outgoing reactions and only include a single species are trivially balanced since the respective species appear in no other complex (for full names of metabolites see caption of Fig. 1). Note that our definition of a trivially balanced complex extends the notion of an intermediate species^30^. In addition, we have shown the prevalence of non-trivially balanced complexes in genome-scale metabolic networks, i.e., there exist balanced complexes that only include species appearing in multiple complexes^17^. In fact, by considering only the steady-state conditions, it is the interlinking of species into complexes that contributes to the formation of balanced complexes.

Next, we say that two complexes, ${C}_{i}$ and ${C}_{j}$, are concordant, if their *activities*, given by ${{\bf{A}}}^{i:}{\bf{v}}$ and ${{\bf{A}}}^{j:}{\bf{v}}$, are coupled, i.e. there exists ${\gamma }_{{ij}}\ne 0$, ${\bf{A}}^{i:}{\bf{v}}-{\gamma }_{{ij}}{\bf{A}}^{j:}{\bf{v}}=0$, for any steady-state flux distribution ${\bf{v}}$ the network obtains^14^. Since the concordance relation is reflexive, symmetric and transitive, it partitions the complexes into equivalence classes called concordance modules^14^. For instance, for the network in Fig. 1A, the concordance modules $\{1\cdot {\rm{Oaa}},\,1\cdot {\rm{AcCoa}}+1\cdot {\rm{Oaa}},\,0\}$, $\{1\cdot {\rm{Succ}},\,1\cdot {\rm{Succ}}+1\cdot {\rm{Gly}},\,1\cdot {\rm{AcCoa}}+1\cdot {\rm{Gly}}\}$, and $\{1\cdot {\rm{AcCoa}}\}$ are represented by different shades of green.

We have already demonstrated that balanced complexes in a set $S$ of steady-state flux distributions can be readily identified by linear programming^18^ (see Methods). The approach amounts to determining that the minimum and maximum activity of a complex are zero across all steady-state flux distributions in the set $S$ of steady-state flux distributions. In previous work, we identified 1.8–58% of the complexes in the analyzed genome-scale metabolic networks to be balanced^18^. In addition, we showed that concordant complexes can also be identified by linear programming that is efficient even for large-scale networks^14^.

In this work, we now ask if additional balanced complexes can be identified in a subset of $S$ obtained by enforcing that a given non-balanced complex, ${C}_{i}$, becomes *forcedly balanced* (by imposing that ${\bf{A}}^{i:}{\bf{v}}=0$). We again stress that the representation of the stoichiometric matrix in terms of species, complexes, and reactions allows us to systematically consider imposing additional balancing constraints guided by the network structure (rather than considering any arbitrary collection of reactions). Further, we denote by ${Q}_{i}$ the set of non-balanced complexes that become balanced by forced balancing of complex ${C}_{i}$; according to this definition, the set ${Q}_{i}$ excludes the complex ${C}_{i}$. The balancing potential of ${C}_{i}$ is then defined by the number of complexes in ${Q}_{i}$. Clearly, a balanced complex ${C}_{i}$ has a balancing potential of zero.

If two non-balanced complexes, ${C}_{i}$ and ${C}_{j}$, are concordant, then ${\bf{A}}^{i:}{\bf{v}}=0$ implies ${\bf{A}}^{j:}{\bf{v}}=0$ and vice versa. Consequently, a complex ${C}_{j}$ that belongs to ${Q}_{i}$ is either concordant or non-concordant to ${C}_{i}$. We say that forced balancing of ${C}_{i}$ *trivially forcedly balances* ${C}_{j}$ if ${C}_{i}$ and ${C}_{j}$ are concordant; otherwise, forced balancing of ${C}_{i}$ *non-trivially forcedly balances* ${C}_{j}$. Therefore, the balancing potential of a non-balanced complex is at least as large as the size of the concordance module to which it belongs minus one. Note that, due to the transitivity and symmetry of the concordance relation, all complexes in a concordance module have the same balancing potential. Further, if forced balancing of ${C}_{i}$ (non-)trivially forcedly balances ${C}_{j}$ then, in the subset of $S$ obtained by imposing ${{\bf{A}}}^{i:}{\bf{v}}=0$, either (I) all reactions around ${C}_{j}$ are blocked, i.e., they are of zero flux, or (II) some incoming and some outgoing reactions from ${C}_{j}$ carry non-zero fluxes. Therefore, the set ${Q}_{i}$ can be partitioned into four parts, namely: complexes that are trivially forcedly balanced of types I or II and non-trivially forcedly balanced of types I or II by forced balancing of ${C}_{i}$. For instance, the balancing potential of the complex $1\cdot {\rm{Oaa}}$ is six, and ${Q}_{{\rm{Oaa}}}$ includes the following complexes: $1\cdot {\rm{AcCoa}},\,1\cdot {\rm{AcCoa}}+1\cdot {\rm{Oaa}},\,1\cdot {\rm{Succ}},\,1\cdot {\rm{AcCoa}}+1\cdot {\rm{Gly}},\,1\cdot {\rm{Succ}}+1\cdot {\rm{Gly}}$, and the zero-complex. Note that of these complexes, $1\cdot {\rm{AcCoa}}+1\cdot {\rm{Oaa}}$ and the zero-complex are trivially forcedly balanced by forced balancing of $1\cdot {\rm{Oaa}}$ as they form a concordance module. The complexes $1\cdot{\rm{AcCoa}},\,1\cdot{\rm{AcCoa}}+1\cdot{\rm{Gly}}$ and $1\cdot{\rm{Succ}}+1\cdot{\rm{Gly}}$ are non-trivially forcedly balanced of type I, while the complex $1\cdot {\rm{Succ}}$ is non-trivially forcedly balanced of type II by forced balancing of complex $1\cdot {\rm{Oaa}}$ (Fig. 1B). Since we are interested in the complexes that are non-trivially forcedly balanced by forced balancing of a given complex, in the following we report the non-trivial forcedly balanced complexes of types I and II, without making the distinction of types for the trivially forcedly balanced complexes.

### Implications of forced balancing in large-scale metabolic networks

**Fig. 2:**
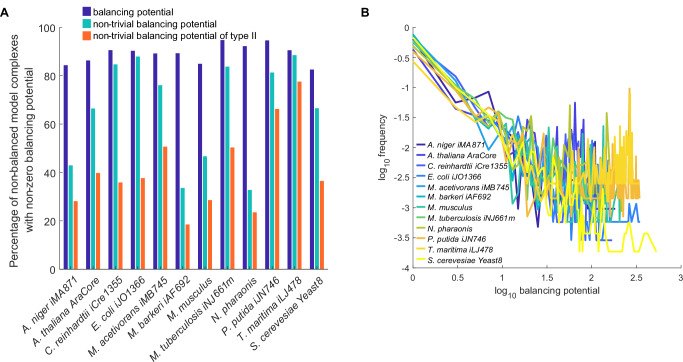
Effects of forced balancing of complexes and distributions of balancing potentials in genome-scale metabolic networks. Fourteen genome-scale metabolic models of twelve organisms from all kingdoms of life are used in the analysis of balancing potential and the consequences of balancing of complexes. **A** Forced balancing of non-balanced complexes leads to balanced complexes across networks. Metabolic networks include all types of balanced complexes resulting from forced balancing of non-balanced complexes. **B** Log-log plot showing the distributions of balancing potential in large-scale metabolic networks. The distributions are best fitted by the power-law with an exponential cut-off.

Having defined and illustrated the concept of forcedly balanced complexes and balancing potential, we were interested in identifying the balancing potential of complexes in real-world biochemical networks. To this end, we made use of genome-scale metabolic networks of twelve organisms from all kingdoms of life, which we had previously used to determine the number of balanced complexes^17,18^ as well as the number and structure of concordance modules^14^. We found that 83–95% of the non-balanced complexes across the analyzed networks exhibited a non-zero balancing potential (Fig. 2A). Moreover, forced balancing of 33–89% of non-balanced complexes across the analyzed models resulted in balancing of complexes outside of the concordance modules to which they belong. Further, forced balancing of 19–78% (on average 41%) of non-balanced complexes across the analyzed models resulted in non-trivial balancing of type II. Thus, all balancing types defined in the previous section could be identified in genome-scale metabolic networks of organisms from all kingdoms of life.

In addition, we investigated the complexes of the highest balancing potential in the analyzed models. The majority of the top ten complexes with the highest balancing potential in each of the models included energy-related metabolites, such as: NADH, NAD, NADP, NADPH, ATP, ADP, and H+ as well as Acetyl-CoA (Supplementary Data S1). This observation, as expected, underlines the prevalence of these metabolites in many reactions and their essentiality for the functioning of metabolic processes. Further, we investigated the pathways related to complexes with high balancing potential using the subsystem information for reactions around the complex. Here, we focused on networks of *Arabidopsis thaliana*, *Chlamydomonas reinhardtii*, *Escherichia coli*, and *Methanosarcina barkeri* for which information on subsystems was available. Complexes with high balancing potential in the model of *A. thaliana* are related to subsystems acetyl-coenzyme A synthesis, glutamine synthesis, α-5’-Phosphoribosyl-1’-pyrophosphat synthesis, pyruvate metabolism as well as carbon fixation. For *C. reinhardtii*, complexes of high balancing potential take part in carbon fixation, fatty acid biosynthesis, oxidative phosphorylation, starch, and sucrose metabolism. In contrast to the two photosynthetic organisms, subsystems with complexes of high balancing potential in *E. coli* and *M. barkeri* were involved in membrane lipid metabolism and the nucleotide salvage pathway as well as alanine-aspartate metabolism and glycine-serine metabolism, respectively. These results indicated that the underlying pathways harboring complexes of high balancing potential are specific to organisms. Moreover, they indicated that the forced balancing of complexes ties together anabolic and catabolic metabolic pathways, imposing further constraints on metabolic functionalities.

### Distribution of balancing potentials in large-scale metabolic networks

Next, we investigated if the distribution of the balancing potentials exhibits similarities across the studied models (Fig. 2B). The balancing potential of complexes that belong to the same concordance module are equal (see above). Previously we showed that the effective degree of metabolites, i.e., the number of concordance modules in which a metabolite participates, is best described by power law and stretched exponential distributions^14^. Therefore, we hypothesized that there are many complexes with low balancing potential and few with a high balancing potential. To test this hypothesis, we fitted the balancing potentials to a power law as well as four non-scale-free distributions, as described by Broido and Clauset^31^. While distributions of balancing potentials cannot be explained by the power-law distribution, we found that all distributions were better described by a power law with exponential cut-off of the form $y={x}^{-a}{e}^{-{bx}}$ with average parameters $a=1.54$ and $b=0.003$ over the different models (p-values for fits to other compared distributions are provided in Supplementary Data S2A)^31^. The parameter $a$ is the power law exponent that scales the tail of the distribution - how common the complexes of high balancing potential are relative to those of low balancing potential. The parameter $b$ introduces a sharp decline for very large values, effectively limiting the frequency of complexes of very high balancing potential.

The values for parameter $a$ vary from 0.86 in *Thermotoga maritima* to 2.16 in *M. barkeri*. The range for parameter $b$ span values of 0.001, for the distributions of balancing potentials in networks of *Aspergillus niger*, *Mus musculus*, *Pseudomonas putida*, and *Saccharomyces cerevisiae*, to 0.007 in *Mycobacterium tuberculosis*. Therefore, the balancing potential across different models belongs to the same class of distributions, denoting a universal property of metabolic networks. Hence, effects imposed by forced balancing of complexes in metabolic networks may provide a new way of altering functionality for various engineering and therapeutic purposes.

### Forcedly balanced complexes in metabolic models of cancer and healthy tissues

To show how metabolic alterations by forced balancing can be used to identify points for intervention, we aimed to investigate whether the metabolic dysregulation in cancerous tissues affects the balancing potential of complexes. If there are complexes with significantly different balancing potentials between healthy and cancerous tissue, their manipulation may offer a way to treat the cancerous tissue while leaving the healthy tissue intact. To examine if forced balancing of complexes can help in the design of new cancer intervention strategy, we compared the balancing potentials of complexes of nine metabolic models of cancerous and corresponding healthy tissues^32^ (Supplementary Fig. S2). The tissue-type specific models of healthy and cancerous cells were obtained by integrating genetic mutation information as well as gene expression profiles into the human metabolic model Recon 2^32^. Therefore, a consistent formulation with respect to stoichiometric scaling is guaranteed across the analyzed healthy and cancer tissue models. In line with observations from the genome-scale models of organisms across kingdoms of life, the distribution of balancing potentials of complexes in metabolic networks of cancer and healthy tissue was best explained by power law with exponential cut-off of the form $y={x}^{-a}{e}^{-{bx}}$. Values for parameter $a$ were in the range from 1.78 to 2.27 for the models of cancerous tissues, with the smallest value found in the model of ovarian carcinoma and the largest value identified for liver hepatocellular carcinoma. The range for the parameter $a$ for the models of healthy tissues was between 1.78 and 2.47 for the gastric tissue model and liver model, respectively. There was no difference between the means of parameter $a$ between cancer and healthy tissue models. However, parameter $b$ was found to be in the range of 0.0001–0.017, with an average value of 0.004, for cancerous tissues and from 0.00001 to 0.03 with an average value of 0.01 for healthy tissues. The smallest value across cancer models was found in the model of kidney carcinoma as well as lung squamous cell carcinoma (SCC), while the largest value was identified for the model of ovarian carcinoma. In healthy tissues the smallest value of parameter $b$ was identified for the model of liver, while the largest value was found for the model of gastric tissue. To further test the significance of the difference in distributions of balancing potentials observed between cancer and healthy tissues, we applied Kolmogorov-Smirnov test. The distribution of balancing potentials was found to be significantly different between all analyzed tissue-specific cancer and healthy models (p-values < 0.01), except in the case of ovarian and lung SCC tissue (p-value 0.07 and 0.94, respectively) (Supplementary Data S2B). These findings indicated that balancing potential exhibits differences between models of healthy and cancerous tissues.

**Fig. 3:**
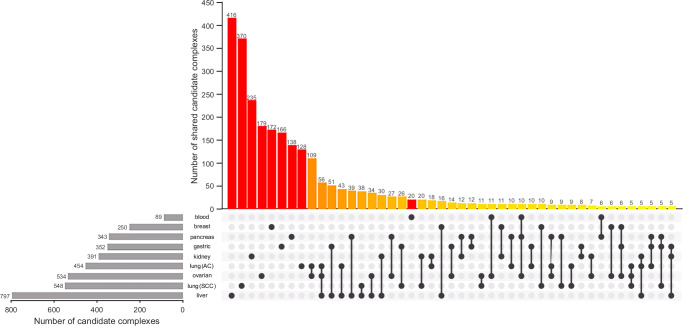
Specificity of cancer interventions based on forced complex balancing. Illustrated is the number of candidate complexes, obtained by forced balancing, per tissue as well as the cardinality of the intersection of candidate complexes between different cancer types. For improved visibility only combinations of cancers that share at least five candidate complexes are presented. The full figure can be found as Supplementary Fig. S2. Tissues and associated cancer (sub)types: blood – acute myeloid leukemia, breast – invasive carcinoma, pancreas – carcinoma, gastric – adenocarcinoma, kidney – renal clear cell carcinoma, lung – adenocarcinoma (AC), lung – squamous cell carcinoma (SCC), ovarian – carcinoma, liver – hepatocellular carcinoma. The vertical bars for the number of candidate complexes specific to individual models of cancer tissues are shown in red. The vertical bars for the number of candidate complexes in the intersection of at least three cancer tissues are shown in orange. The gray horizontal bars indicate the total number of candidate complexes in the respective cancer tissue.

Importantly, we found a change in the balancing potential between cancer and the corresponding healthy tissue for some of the complexes that were present in both models across the different tissue types. For instance, this was the case for 28–47% of complexes shared between models of cancer and corresponding healthy tissues, respectively. These changes in balancing potential of complexes between cancerous and healthy tissue models may allow the identification of interventions that are harmful to cancerous but can be tolerated by healthy cells. To test this hypothesis, we made use of the biomass reaction part of the metabolic models of healthy and cancer tissues and investigated complexes whose forced balancing was found to be lethal, i.e., lead to no growth, in the cancer tissue model, but allowed at least 90% of the optimal growth in the corresponding healthy tissue model, deduced by flux balance analysis (FBA) (see Methods). Note that although optimization of growth might not be the objective fulfilled by human cells, the described procedure can accommodate any other suitable objective function. We could identify between 89 (blood) and 797 (liver) complexes whose forced balancing was lethal in the cancer tissue model, but not in the corresponding healthy tissue model (Fig. 3, horizontal bars). We observed that there was no complex whose forced balancing was lethal in all cancer models, yet without an effect on growth in all models of healthy tissues. However, we found three complexes, namely $1\cdot {\rm{retinol}}-9-{\rm{cis}}[{\rm{e}}],\,1\cdot {\rm{retinol}}-{\rm{cis}}-11[{\rm{e}}]$, and $1\cdot {\rm{lys}}-{\rm{L}}[{\rm{e}}]$, whose balancing was lethal for cancer but not for healthy tissue models, namely: pancreas, gastric, lung (adenocarcinoma (AC)), ovarian, and liver (Supplementary Data S3, Supplementary Fig. S2). Altogether, we found that 21 pairs of cancer types shared between 10 and 109 *candidate complexes* whose forced balancing was lethal in cancer tissues but not in the corresponding healthy tissue model; moreover, there were only two triplets of cancer tissues that shared at least ten0 such candidate complexes (Fig. 3). These finding indicates that there are substantial differences in the balancing potential between cancer and healthy tissue models, despite similarities in the metabolic networks. The candidate complexes were, interestingly, largely cancer-type specific (Fig. 3, red bars), ranging from 23% of the candidate complexes in acute myeloid leukemia to 68% in breast cancer model. These findings further support the claim about the specificity of interventions that can mitigate growth of cancer tissues without an effect on the corresponding healthy tissue.

To test the relevance of forced balancing of complexes as a mechanism for cancer intervention strategies, we asked to what extent the lethality of forcedly balanced complexes was associated with the essentiality of their surrounding reactions. Here, we note that blocking of essential reactions is lethal, i.e., abolishes growth in the analyzed model^20,33^. We found that in the cancer tissue models, between 6 and 49% of the reactions in the concordance module of a candidate complex whose balancing was lethal were essential. In addition, we found that concordance modules of lethal balancing complexes were enriched for essential reactions for all tissue types (Fisher exact test, p-value < 0.01). In line with this observation, the number of lethal balancing complexes correlates with the number of reactions switching from non-essential in healthy tissues to essential in the corresponding cancerous tissue (Pearson correlation 0.88, p-value 0.002). In contrast, the general difference in the number of essential reactions in healthy and corresponding cancerous tissue is weakly correlated with the number of lethal balancing complexes (Pearson correlation 0.28, p-value = 0.46). However, we also identified that concordance modules of 0.6–52.5% of the candidate complexes did not include any essential reactions (Supplementary Data S4A). Therefore, our results indicate that lethality induced by forcedly balanced complexes can help in identifying radically new points for intervention in cancer metabolism, with minimal effects on growth predicted in healthy tissues with respect to default flux bounds.

## Discussion

Recent advances in the analysis of how the structure of metabolic networks together with physico-chemical constraints affect metabolic phenotypes^14,16–18,34^ have contributed to better understanding of the complexity of metabolic networks. However, while these advances have facilitated feasible partitioning of metabolic networks into modules using the notion of multireaction dependencies and relations to network structure, they shed no light on manipulations of metabolic functions. Here, we ask how imposing multireaction dependencies affects balancing in specific points of a metabolic network, defined by the complexes of the network. This prompted us to define and investigate the notion of balancing potential of a complex, how this notion relates to concordant modules, and growth supported by a metabolic network.

Interestingly, we showed that the balancing potential follows power-law distribution with exponential cut-off across metabolic models of different organisms as well as different tissue types. The scale-freeness with cut-off is typical for networks of finite size^35^. While the scaling of balancing potentials appears as a universal property of real-world metabolic models, the complexes with the largest balancing potential were not shared across species. These may result from the differences in the underlying metabolic network models and the particularities of their reconstructions. However, all these complexes included energy currency metabolites, indicating that the balancing potential is related to the participation of metabolites in large number of complexes and reactions. To a certain extent these findings are observation parallel to the seminal results about scale-freeness of metabolic networks^36^, obtained by graph representation of the hypergraph structure of metabolic networks^37^. We note that the complex-centric representation of metabolic networks used in our work does not remove any currency metabolites (e.g., cofactors, water, protons) in arriving of these findings. In addition, the balancing of complexes, in contrast to degree distributions, is a functional property that is obtained by imposing steady-state and other physicochemical constraints.

Regarding biotechnological applications of the concept of balancing, we compared and contrasted the balancing potential between models of cancer and healthy tissues. Surprisingly, we identified candidate complexes whose forced balancing was lethal in the cancer, but had minimal effect on growth in healthy tissue models. While growth might not be the objective healthy human cells maximize, the same workflow can be applied to refined metabolic networks considering another objective function in the future. In addition, these complexes were largely specific to particular cancer tissues. However, the feasibility of this strategy depends of whether the balancing of a complex can be imposed by simple network modifications.

We note that a complex can be forcedly balanced by knocking out the genes underlying the reactions incident on the complex. This form of balancing, however, may not be feasible due to either gene conflicts^38^ or the need to identify inhibitors specific to enzymes underlying the reactions incident on the complex^39,40^. The latter strategy may also have detrimental effects in both cancer and healthy cells, due to the shared essentiality of reactions in these models. We already established that a complex is trivially balanced if it contains a species that does not appear in any other complex in the network. Therefore, one way that renders balancing of a non-balanced complex is to modify its incident reactions, allowing for inclusion of a new species that is not already present in the network (Supplementary Fig. S3). However, this modification requires extensive enzyme engineering and is not practically applicable. Another strategy by which a non-balanced complex can be balanced is by modifying the model to include an exchange reaction, which can be used to balance the activity of the complex. This can be readily achieved for those complexes that include only internal species, which we here define as species for which no exchange reactions are present in the underlying metabolic network (Supplementary Data S4A,B). Note that, for sink and source complexes the balancing potential observed forced balancing by addition of an exchange reaction can differ from the results obtained by imposing the constraint ${{\bf{A}}}^{i:}{\bf{v}}=0$, since addition of an exchange flux will not block reactions around the sink or source complex. In practice, this type of modification entails identifying or engineering a transporter for the metabolite included in the complex that we aim to balance; in the case of cancer mitigation strategies, the transporter will be expressed in all cells, further contributing to the feasibility of complex balancing in controlling growth of cancer and healthy cells. For instance, recent metabolic mapping of transport proteins can be used a step to rendering this strategy experimentally feasible^41^. The simplest case is given by forced balancing complexes that include a single internal metabolite. Possible candidates that were identified in up to four tissue types include metabolites, such as: glycerol-3-phosphate, 10-formyltetrahydrofolate, energy metabolism related metabolites like ADP, NAD and NADP, propanoyl-CoA, 13-cis-retinoate, arachidonate, bilirubin and taurocholic acid. Interestingly, majority of these metabolites are known for their dysregulation^42,43^ or promotion of cancer^44^ and have been suggested as possible cancer treatment^45–48^. However, we note that the transport of these metabolites may lead to changes in other system’s functions that still have to be investigated. Altogether, our results indicated that balancing of complexes represents a feasible, radically new strategy in the fight against cancers.

Since the presented concepts rely on techniques from convex optimization, future work can analyze the effect of additional constraints, related to allocation of enzyme resources^49,50^, in the context of balancing. Moreover, the work paves the way for systematic investigation and usage of multireaction dependencies in the design of metabolic engineering strategies for various biotechnological applications.

## Methods

### Models and their processing

The genome-scale metabolic models of twelve organisms (Supplementary Data S1), were obtained from their original publications^51–62^. The blocked reactions, reactions with absolute flux values less than 10^-9^ ${mmolgD}{W}^{-1}{h}^{-1}$, in the metabolic network were determined by Flux Variability Analysis^63^ and were removed from the original models, as performed in other studies^14,16–18,64^. Each reversible reaction was split into two irreversible reactions. The lower bounds for irreversible reactions were set to zero, while the upper bounds were fixed to the maximum of the upper bounds in the original model. Optimum biomass was determined per FBA^65^.

### Identification of balanced complexes

Let ${\bf{Y}}$ denote the non-negative matrix of complexes, with rows denoting species and columns representing complexes. The entry ${y}_{{ij}}$ denotes the stoichiometry with which species $i$ enters the complex ${C}_{j}$. Let ${\bf{A}}$ denote the incidence matrix of the directed graph with nodes representing complexes and edges denoting reactions. The rows of ${\bf{A}}$ denote the complexes and its columns stand for the reactions. Since the graph is directed, each column of ${\bf{A}}$ has precisely one -1 and one 1 entry, corresponding to the substrate and product complexes of the respective reaction. The stoichiometric matrix is then given by ${\bf{N}}={\bf{YA}}$.

The sum of fluxes around the complex ${C}_{j}$ is given by ${\bf{A}}^{j:}{\bf{v}}$. A complex is balanced in the set of flux distributions $S=\left\{{\bf{v}}|{\bf{Nv}}=\bf 0,\,{\bf{v}}_{\min }\le {\bf{v}}\le {\bf{v}}_{\max }\right\}$ if ${\bf{A}}^{j:}{\bf{v}}=0$ for every ${\bf{v}}\in S$. This condition can be verified by determining that the minimum and maximum values of ${\bf{A}}^{j:}{\bf{v}}$ equal to 0. The latter can be ensured by solving two linear programs:

$$\min /\max {\bf{A}}^{j:}{\bf{v}}$$

s.t.

$${\bf{YAv}}={\bf{Nv}}={\bf{0}}$$

$${\bf{v}}_{\min }\le {\bf{v}}\le {\bf{v}}_{\max }$$

Checking that complex ${C}_{j}$ becomes balanced by balancing of complex ${C}_{i}$ imposes expansion of the constraints in the linear program(s) above; namely:

$$\max /\max {{\bf{A}}}^{j:}{\bf{v}}$$

s.t.

$${\bf{YAv}}={\bf{Nv}}={\bf{0}}$$

$${\bf{A}}^{i:}{\bf{v}}=0$$

$${\bf{v}}_{\min }\le {\bf{v}}\le {\bf{v}}_{\max }.$$

Note that information about concordance modules can speed up the calculations, since balancing of any complex renders all complexes in its concordance module balanced.

### Predictions of lethality in cancer and healthy tissue models

We calculate optimal specific growth rates, $z$, by FBA in models of cancer and healthy tissues^32^. The tissue-type specific models generated by Nam et al^32^. of healthy and cancerous cells were originally obtained by integrating genetic mutation information as well as tissue-specific gene expression profiles into generic models of human metabolism. The optimal specific growth rate when balancing complex ${C}_{i}$, ${{\rm{z}}}_{{\rm{i}}}^{* }$, was obtained by solving the following linear program:

$${{\rm{z}}}_{{\rm{i}}}^{* }=\mathrm{max}\,{{\rm{v}}}_{{\rm{bio}}}$$

s.t.

$${\bf{YAv}}={\bf{Nv}}={\bf{0}}$$

$${\bf{A}}^{i:}{\bf{v}}=0$$

$${\bf{v}}_{\min }\le {\bf{v}}\le {\bf{v}}_{\max }.$$

Balancing of complex ${C}_{i}$ is considered lethal for ${{\rm{z}}}_{{\rm{i}}}^{* } < 1{\rm{e}}-9$.

## Supplementary information

Supplementary Information

Supplementary_Table_S1

Supplementary_Table_S2

Supplementary_Table_S3

Supplementary_Table_S3

## Data Availability

The approach is implemented and available together with all data needed to reproduce the findings at [https://github.com/ankueken/forced_balancing].
