# Supplementary material for: A constraint-based framework for exploring the impact of multireaction dependencies on metabolic functions
Source: NPJ Syst Biol Appl. 2025 Oct 23;11:118. doi: 10.1038/s41540-025-00608-9 (PMC12549881; doi:10.1038/s41540-025-00608-9)
Supplement: Supplementary file 1 — Supplementary Information [file 41540_2025_608_MOESM1_ESM.pdf]

Supplementary Figures

a

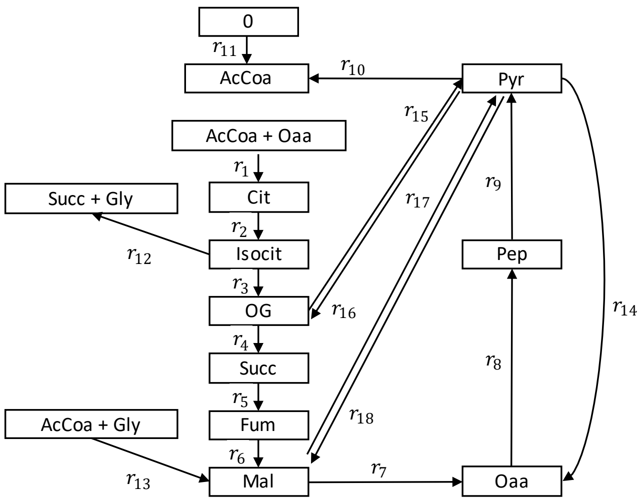

b

$Y =$

	AcCoA	AcCoA + Gly	AcCoA + Oaa	Cit	Fum	IsoCit	Mal	Oaa	OG	Pep	Pyr	Succ	Succ + Gly	0
AcCoA	1	1	1	0	0	0	0	0	0	0	0	0	0	0
Cit	0	0	0	1	0	0	0	0	0	0	0	0	0	0
Fum	0	0	0	0	1	0	0	0	0	0	0	0	0	0
Gly	0	1	0	0	0	0	0	0	0	0	0	0	1	0
IsoCit	0	0	0	0	0	1	0	0	0	0	0	0	0	0
Mal	0	0	0	0	0	0	1	0	0	0	0	0	0	0
Oaa	0	0	1	0	0	0	0	1	0	0	0	0	0	0
OG	0	0	0	0	0	0	0	0	1	0	0	0	0	0
Pep	0	0	0	0	0	0	0	0	0	1	0	0	0	0
Pyr	0	0	0	0	0	0	0	0	0	0	1	0	0	0
Succ	0	0	0	0	0	0	0	0	0	0	0	1	1	0

c

$A =$

	r_1	r_2	r_3	r_4	r_5	r_6	r_7	r_8	r_9	r_{10}	r_{11}	r_{12}	r_{13}	r_{14}	r_{15}	r_{16}	r_{17}	r_{18}
AcCoA	0	0	0	0	0	0	0	0	0	1	1	0	0	0	0	0	0	0
AcCoA + Gly	0	0	0	0	0	0	0	0	0	0	0	0	−1	0	0	0	0	0
AcCoA + Oaa	−1	0	0	0	0	0	0	0	0	0	0	0	0	0	0	0	0	0
Cit	1	−1	0	0	0	0	0	0	0	0	0	0	0	0	0	0	0	0
Fum	0	0	0	0	1	−1	0	0	0	0	0	0	0	0	0	0	0	0
IsoCit	0	1	−1	0	0	0	0	0	0	0	0	−1	0	0	0	0	0	0
Mal	0	0	0	0	0	1	−1	0	0	0	0	0	1	0	0	0	−1	1
Oaa	0	0	0	0	0	0	1	−1	0	0	0	0	0	1	0	0	0	0
OG	0	0	1	−1	0	0	0	0	0	0	0	0	0	0	−1	1	0	0
Pep	0	0	0	0	0	0	0	1	−1	0	0	0	0	0	0	0	0	0
Pyr	0	0	0	0	0	0	0	0	1	−1	0	0	0	−1	1	−1	1	−1
Succ	0	0	0	1	−1	0	0	0	0	0	0	0	0	0	0	0	0	0
Succ + Gly	0	0	0	0	0	0	0	0	0	0	0	1	0	0	0	0	0	0
0	0	0	0	0	0	0	0	0	0	0	−1	0	0	0	0	0	0	0

Supplementary Figure S1. Illustration of concepts related to balancing of complexes. (**a**) Network including 11 species (AcCoA, - Acetyl-CoA, Cit – Citrate, Fum – Fumarate, Gly - Glyocalate, Isocit – Isocitrate, Mal – Malate, Oaa – Oxaloacetate, OG – Oxoglutarate, Pep – Phosphoenolpyruvate, Pyr – Pyruvate, Succ – Succinate, 0 – zero-complex), 14 complexes including the zero-complex that models the interaction with the environment, depicted as rectangles, and 18 irreversible reactions, $r_1 - r_{18}$, each connecting two complexes. (**b**) Species-complex matrix **Y** of the network in (**a**), where rows correspond to species and column correspond to complexes. Each entry indicates the molarity with which a species participates in a complex. (**c**) Incidence matrix **A** of the directed graph given in (**a**). The stoichiometric matrix of the network is then given by the product of the species-complex matrix and the incidence matrix, $\mathbf{N} = \mathbf{Y} \cdot \mathbf{A}$.

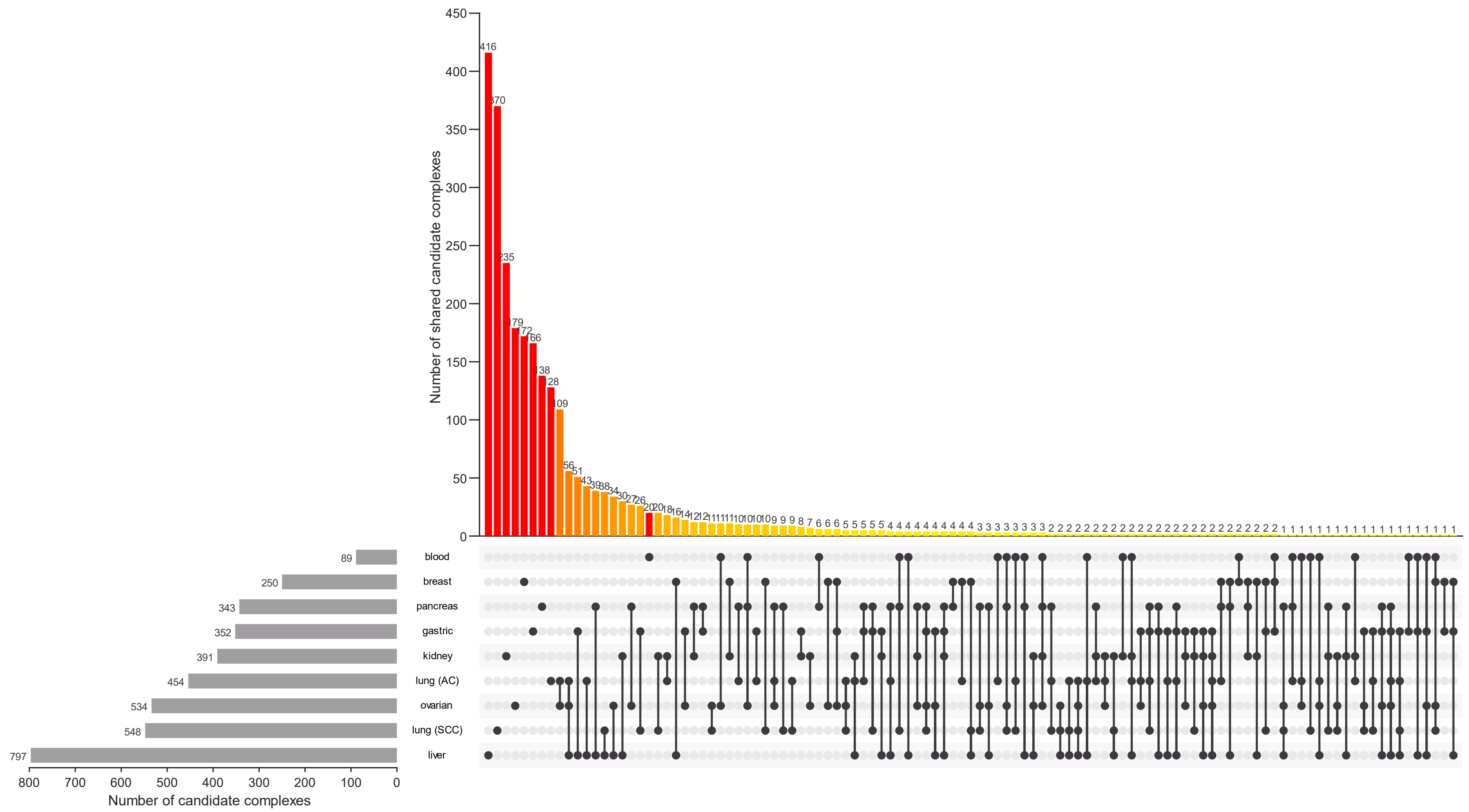

Supplementary Figure S2. Specificity of cancer interventions based on complex balancing. Illustrated is the number of candidate complexes, obtained by forced balancing, per tissue as well as the cardinality of the intersection of candidate complexes between different cancer types. Tissues and associated cancer (sub)types: blood – acute myeloid leukemia, breast – invasive carcinoma, pancreas – carcinoma, gastric – adenocarcinoma, kidney – renal clear cell carcinoma, lung – adenocarcinoma (AC), lung – squamous cell carcinoma (SCC), ovarian – carcinoma, liver – hepatocellular carcinoma. The bars for the number of candidate complexes in individual models of cancer tissues are shown in red.

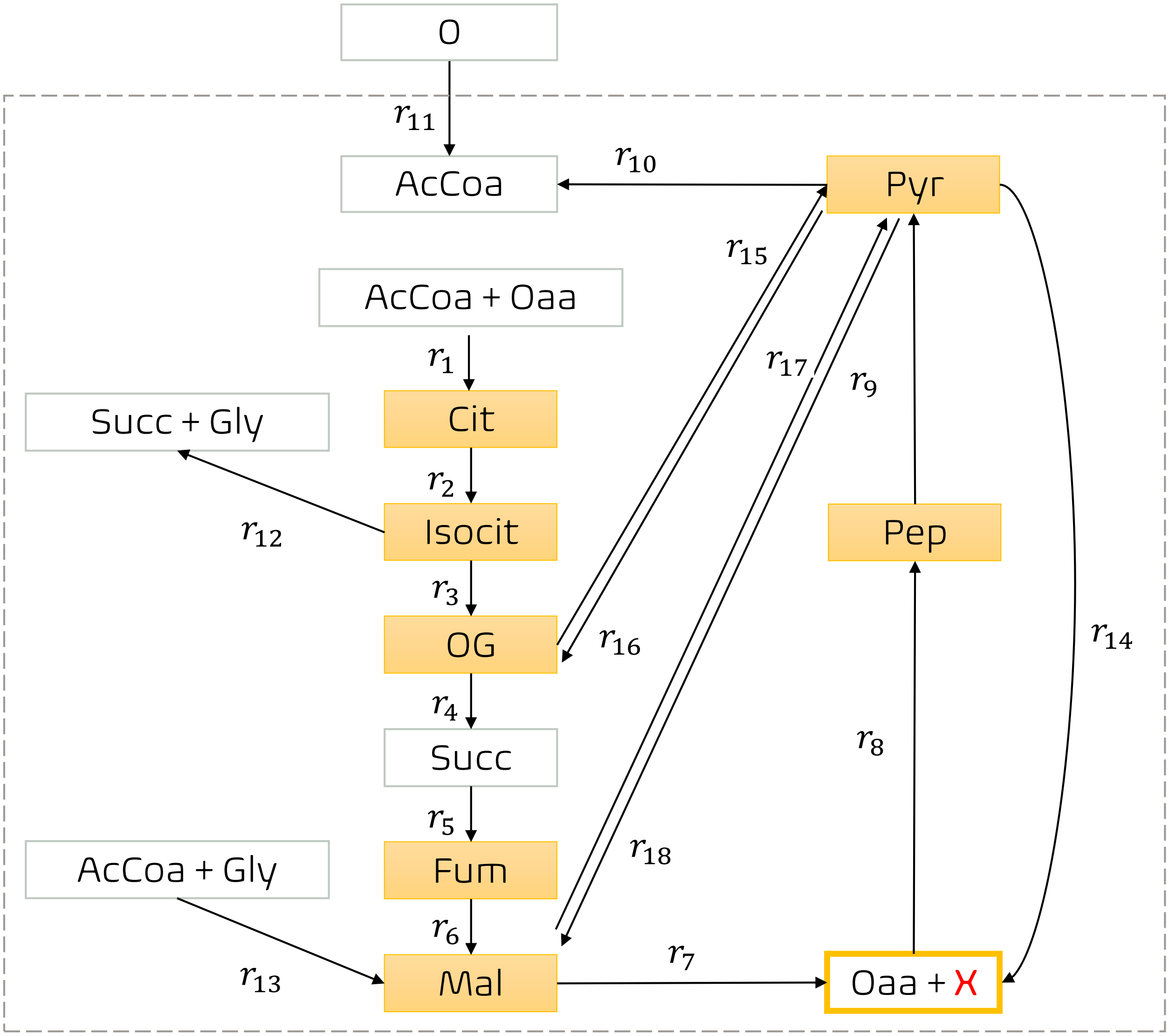

Supplementary Figure S3. Illustration of balancing complexes by inclusion of phantom-species. The complex $1 \cdot \text{Oaa}$ being unbalanced in the original example network (see Supplementary Figure S1) becomes balanced by introducing phantom species X. The resulting complex $1 \cdot \text{Oaa} + 1 \cdot \text{X}$ is trivially balanced as species X does not appear in any other complex in the network. Balanced complexes are shown in yellow. The balancing complex is marked by a yellow box. Metabolite abbreviations: AcCoA, - Acetyl-CoA, Cit – Citrate, Fum – Fumarate, Gly - Glyocalate, Isocit – Isocitrate, Mal – Malate, Oaa – Oxaloacetate, OG – Oxoglutarate, Pep – Phosphoenolpyruvate, Pyr – Pyruvate, Succ – Succinate, 0 – zero-complex.

Supplementary Data Captions

Supplementary Table S1. Top 10 complexes with highest balancing potential across twelve organisms from all kingdoms of life.

Supplementary Table S2. Parameter values that best fit balancing distributions of organism-specific networks for four different distributions. The provided p-values are based on two-sample Kolmogorov-Smirnov test. As described in Broido et al. (Nat Comms. 2019) fit of alternative distributions in comparison to power law are evaluated using likelihood-ratio test (1 - power law better, 0 - inconclusive, -1 - alternative dist better).

Supplementary Table S3. Complexes whose balancing is lethal in the model of cancer tissue, but allows at least 90% of optimal growth in the respective healthy tissue model. Compartment abbreviations: Cytoplasm - [c], Extracellular - [e], Golgi - [g], Lysosome - [l], Mitochondria - [m], Nucleus - [n], Endoplasmic reticulum - [r], Peroxisome - [x]

Supplementary Table S4. (**A**) Candidate complexes whose balancing is lethal in cancerous tissue, but not in healthy tissue and whose concordance module does not include essential reactions in the cancer model. We suggest that, these complexes can be balanced by transporter engineering. Therefore, complexes that contain a metabolite already exchanged with the environment are excluded from the presented list. Note that no candidate complex without metabolites being exchanged could be found for gastric cancer. (**B**) Metabolites part of candidate complexes (see Table S4A) whose balancing is lethal in cancerous tissue, but not in healthy tissue and whose concordance module does not include essential reactions in the cancer model. (**C**) Metabolites part of candidate complexes (see Supplemental Table S4A) that contain a single metabolite only, whose balancing is lethal in cancerous tissue, but not in healthy tissue and whose concordance module does not include essential reactions in the cancer model.
